# A Protein with Unknown Function, Ps495620, Is Critical for the Sporulation and Oospore Production of *Phytophthora sojae*

**DOI:** 10.3390/jof11010012

**Published:** 2024-12-27

**Authors:** Xiaoran Du, Yan Zeng, Yiying Li, Qin Peng, Jianqiang Miao, Xili Liu

**Affiliations:** 1 State Key Laboratory for Crop Stress Resistance and High-Efficiency Production, College of Plant Protection, Northwest A&F University, Yangling, Xianyang 712100, China; duxiaoran@nwafu.edu.cn (X.D.); zengy202021@163.com (Y.Z.); liyiying036@163.com (Y.L.); pengqin1991@126.com (Q.P.); 2 Department of Plant Pathology, College of Plant Protection, China Agricultural University, 2 Yuanmingyuanxi Road, Beijing 100193, China

**Keywords:** *Phytophthora sojae*, CRISPR/Cas9, gene function, zoospore, oospore

## Abstract

While the rapid rise in bioinformatics has facilitated the identification of the domains and functions of many proteins, some still have no domain annotation or largely uncharacterized functions. However, the biological roles of unknown proteins were not clear in oomycetes. An analysis of the *Phytophthora sojae* genome database identified the protein Ps495620, which has no domain annotations and functional predictions in *Phytophthora*. This study used a CRISPR/Cas9-mediated gene replacement system to knock out *Ps495620* to elucidate its function. The *Ps495620*-knockout mutants exhibited significantly increased oospore production and decreased sporangium formation compared to the wild-type strain P6497. Transcriptomics showed that it is a key regulator of nitrogen, pyruvate, ascorbate, and adorate metabolism in *P. sojae*. Our findings indicate that Ps495620 is critical in regulating sporangium formation and oospore production in *P. sojae*.

## 1. Introduction

*Phytophthora sojae* is a significant soil-borne plant pathogenic oomycete, recognized among the top ten plant pathogenic oomycetes worldwide, causing substantial economic losses in soybean production annually [1]. As a hemibiotrophic oomycete, *P. sojae* reproduces sexually through syngamy, resulting in the production of a large number of oospores, while asexual reproduction leads to the formation of sporangia and the release of zoospores. These zoospores rapidly differentiate into cysts, which then germinate into mycelium. *Phytophthora sojae* primarily infects its host through zoospores [2]. Additionally, resting spores form on the surface of plant roots, and the germ tubes they produce can infect through the roots. Zoospores and sporangia can also be spread to aerial parts of plants by splashing water.

The complete genome sequence of *P. sojae* has been published, and it has gradually become a model species for studying plant pathogenic oomycetes [1]. With the development of gene editing technologies, CRISPR/Cas9 has become an effective tool for studying gene function in oomycetes [3]. However, current research on the signaling pathways and key proteins involved in both the sexual and asexual reproduction of plant pathogenic oomycetes, such as *P. sojae*, remains relatively limited.

Sexual reproduction is a crucial aspect of the oomycete life cycle, involving the production of thick-walled oospores that withstand adverse conditions and maintaining genetic diversity within the population. The oospore accumulates lipids, proteins, and β-glucans and forms a multilayered cell wall, which provides high environmental tolerance and supports long-term survival [4]. Multiple genes regulate various stages of development and function in the sexual reproduction of *Phytophthora* spp. The *PsCZF1* gene, which encodes a C_2_H_2_ zinc finger protein, is crucial in oospore development and pathogenicity. Experiments have shown that silencing *PsCZF1* results in impaired oospore formation and significantly reduced pathogenicity [5]. The *PlLLP* gene is also important in oospore formation and plant infection, with its silencing leading to reduced oospore formation and pathogenicity [6]. Receptor-like kinases such as PsRLK6 not only regulate oospore maturation but also activate plant pattern-triggered immunity, indicating that the pathogen’s reproductive regulation is linked to host interactions [7].

Asexual reproduction is vital for the rapid spread of *P. sojae*, involving sporangia and zoospores as the primary vectors. Temperature and humidity are critical environmental factors influencing sporangium formation. Under high humidity, sporangia cleave to release zoospores [8]. Nutrient limitation and water exposure have also been shown to induce sporangium development in *P. sojae* [9]. Sporangia development and subsequent zoospore release involve complex genetic regulation. In *P. sojae*, the TatD nuclease family plays a significant role in sporulation. PsTatD4 has DNase activity linked to programmed cell death and is upregulated during sporangium formation and the later stages of infection [10]. Transcription factor PsMAD1 is essential for the formation and cleavage of sporangial cytoplasm into zoospores. Knockout studies of *PsMAD1* revealed normal sporangia production but failed zoospore release, indicating its pivotal role in zoospores [11]. G protein-mediated pathways are implicated in fungal and oomycete sporulation processes. They link environmental cues to developmental responses, impacting both zoospore release and spore germination [12]. While several key genes involved in the sexual and asexual reproduction of *P. sojae* have been identified, understanding their regulatory networks and signaling pathways remains an important task for future research.

Unknown proteins have been discovered, and their functions and domains remain unclear. These proteins are widely present across various organisms, and studying them is crucial for understanding biological processes, discovering new functional proteins, and developing therapeutic methods. Furthermore, advances in sequencing technologies have led to the accumulation of genomic, transcriptomic, and proteomic data, revealing proteins with domains of unknown function (DUF) in the Pfam database [13]. These proteins, which possess relatively conserved amino acid sequences, are categorized as members of the DUF family. DUF proteins are provisionally named with the prefix “DUF” followed by a number. Once their functions are elucidated, they are renamed or merged with other well-known domain families [14]. Many studies have identified DUF-containing proteins across various organisms, including bacteria [15], plants [16], animals [17], and protists [15]. DUF domains are involved in the synthesis and modification of cell walls [18], reproductive organ development [19], biotic stress resistance [20], abiotic stress tolerance [21], and the ABA (abscisic acid) signaling pathway [22]. Interestingly, proteins without known domains, signals, and motifs still regulate vital activities found in many species. In *Puccinia striiformis*, Pst11215 contains no known domains but manipulates mitochondria to suppress host immunity by promoting TaVDIP1-mediated ubiquitination of TaVDAC1 [23]. In *Fusarium graminearum*, FgEC1 targets wheat TaGF14b to suppress TaRBOHD-mediated reactive oxygen species production and promote infection [24]. In *Puccinia striiformis*, Pst03724 modulates host immunity by inhibiting NAD kinase activation by calmodulin [25].

Despite the identification of numerous unknown proteins across various species, their biological functions in the asexual and sexual reproduction of *P. sojae* remain unexplored. In this study, we identified a protein with an unknown function, Ps495620 (PHYSODRAFT_495620), in *P. sojae*. Modifications to the Ps495620 sequence were made using RNAseq data from FungiDB to proofread the protein’s full-length sequence. We used the CRISPR/Cas9 system to confirm that Ps495620 is critical for the sporulation and oospore production of *P. sojae* and preliminarily analyzed Ps495620-regulated biological signaling pathways via transcriptomics.

## 2. Materials and Methods

### 2.1. Sequence and Phylogenetic Analyses of Ps495620

The gene sequences of *Ps495620* (PHYSODRAFT_495620) were retrieved from the FungiDB database (https://fungidb.org/fungidb/app). Modifications to the Ps495620 sequence were made using RNAseq data from FungiDB and cDNA verified by sequencing. Homologous proteins were identified and analyzed using NCBI search tools (https://www.ncbi.nlm.nih.gov/search/). Multiple sequence alignments of Ps495620 homologs were initially created using the Muscle algorithm (MEGA software, version 11.0.13). A phylogenetic tree was constructed using Bayesian inference with MEGA software (version 11.0.13) based on 1000 bootstraps replications.

### 2.2. Phytophthora sojae Strains and Culture Conditions

This study used a wild-type (WT) strain of *P. sojae* (P6497), which was provided by Dr. Brett M. Tyler (Oregon State University). It was cultured on 10% V8 juice (V8 Original 100% Vegetable Juice, Bedford Park, IL, USA) agar at 25 °C in a dark incubator (NOKI, Changzhou, China). Growth assays used Plich medium containing 0.5 g KH_2_PO_4_ (Sigma, Steinheim, Germany), 0.5 g yeast extract (OXOID, Basingstoke, UK), 1 g asparagine (Sigma, Germany), 0.25 g MgSO_4_·7H_2_O (Sigma, Germany), 1 mg thiamine (Sigma, Germany), 25 g glucose (Sigma, Germany), 10 mg β-sitosterol (Sigma, Germany), and 15 g agar (Solarbio, Beijing, China). Additionally, 0.015% H_2_O_2_, 0.5 M KCl (Sigma, Germany), or 1 M sorbitol (Sigma, Germany) were added as required in the stress tests of transformants [26]. The soybean cultivar Williams was grown in substrate (Pindstrup, Bremerhaven, Germany) in a glasshouse at 28 °C under a 16/8 h light/dark photoperiod.

### 2.3. RNA Extraction and Gene Expression Analysis of Ps495620

Samples were collected from different developmental stages of *P. sojae*: mycelium (MY), sporangium (SP), zoospore (ZO), cystospore (CY), cyst germination (CG), and various infection stages of 0.1 g mycelium of P6497 strain (at 1.5, 3, 6, 12, 24, and 48 h) on soybean leaves [27]. Total RNA was extracted using the SteadyPure Plant RNA Extraction Kit (Accurate Biology, Changsha, China). First-strand cDNA was synthesized from 1 µg total RNA using the Evo M-MLV RT Kit with gDNA Clean for qPCR II (Accurate Biology, Changsha, China). Quantitative real-time PCR (qRT-PCR) was performed on a Bio-Rad CFX Connect Real-Time PCR System (Hercules, CA, USA) using Tsingke TSE501 ArtiCanATM SYBR qPCR Mix (Tsingke Biotechnology, Beijing, China). The *Actin* gene from *P. sojae* was used according to Li et al. [28], and the primers used in this experiment are listed in Table S1. Relative *Ps495620* transcript levels were calculated using the 2^−∆∆CT^ method [29]. Means and standard deviations were obtained from three biological replicates.

### 2.4. Plasmid Construction

Gene knockout transformants were generated using the CRISPR/Cas9-mediated gene editing system [3]. The single-guide RNA (sgRNA) was designed using the EuPaGDT web tool (http://grna.ctegd.uga.edu) and cloned into the pYF515 plasmid [3]. The 1 kb sequences upstream and downstream of the target gene were amplified and cloned into the pBS II KS+ donor vector, with the *GFP* gene replacing the target gene. The sgRNAs and primers used in this experiment are listed in Table S1.

### 2.5. Phytophthora sojae Transformation

Gene knockout transformants were generated using the polyethylene glycol-mediated protoplast transformation method, as described previously [3]. Positive transformants were selected on a V8 medium containing 50 µg/mL G418 (G-418 sulfate, geneticin) and verified by PCR. The primers used in this part are also listed in Table S1. A complemented transformant was also selected on a V8 medium containing 50 µg/mL G418.

### 2.6. Phenotype Analysis of Transformants and P. sojae

Mycelial growth: A 5 mm plug was inoculated onto 10% V8 agar and incubated at 25 °C in the dark incubator (NOKI, China) for six days. Colony diameter was measured with a vernier caliper by two cross measurements. Sensitivity to various temperatures (4 °C, 13 °C, 18 °C, 25 °C, 30 °C, and 37 °C), osmotic stress, and oxidative stress was tested by culturing P6497 and the transformants on Plich medium in the dark.

Sporangial production: P6497 and the transformants were cultured on V8 plates at 25 °C in the dark incubator (NOKI, China) for six days. Plates were washed with sterile water seven times every 20 min, and 5 mL of water was added to the plates, followed by incubation at 25 °C for three hours in the room. One third of distance from the mycelium edge to center of the whole plate was chosen, and five visual fields of microscopy were randomly selected at 10× magnification; sporangia were counted at different levels of the same visual field to quantify the number of sporangia using microscopy (Nikon, Tokyo, Japan).

Zoospore production: The P6497 and transformants were cultivated on 10% V8 plates for six days at 25 °C in the dark incubator (NOKI, China). The production of zoospores was induced by washing the plates with distilled water seven times, then adding 5 mL sterile water to the plates and incubating the isolates at 25 °C in the dark for four hours. Zoospore production was quantified with a hemacytometer (Qiu Jing, Shanghai, China).

Cyst germination: Zoospores were collected in a 50 mL centrifuge tube, and cysts were generated by vortexing for 30 s. Cyst germination was observed using microscopy after overnight incubation on water agar at 25 °C in the dark incubator (NOKI, China).

Oospore production: P6497 and the transformants were cultured on 10 mL 10% V8 juice agar in 90 mm dishes. V8 plates were left at 25 °C in the dark for seven days. Two thirds of distance from the mycelium edge to center of the whole plate was chosen, five visual fields of microscopy were randomly selected at 10× magnification, and oospores were counted at different levels of the same visual field to quantify the number of oospores using microscopy (Nikon, Japan).

Pathogenicity: A 5 mm mycelial plug was used to inoculate the first true leaves of 10-day-old soybean plants (cultivar: Williams, substrate: Pindstrup, Germany). After 48 h of incubation, photos were taken under UV light, and lesion areas were analyzed using the ImageJ software (version 2.14.0/1.54j).

All the above phenotype experiments were repeated at least three times.

### 2.7. Transcriptome Sequencing and Analysis

RNA-seq was performed to investigate changes in gene expression profiles in P6497 and *Ps495620* knockout transformants. Cultures were grown in 10% liquid V8 medium for three days, and mycelia were washed with sterile water seven times every 20 min before collecting mycelia with sporangia three hours later. The 0.3 g material was dried using a vacuum filter pump (Ruicheng Weiye, Beijing, China) and stored in 1.5 mL tubes at −80 °C (Thermo, Waltham, MA, USA) until analyzed. Three biological replicates were performed for P6497 and ∆Ps495620-1 mutants. RNA integrity was assessed using a Bioanalyzer 2100 system (Agilent Technologies, Santa Clara, CA, USA). The cDNA libraries were sequenced on an Illumina NovaSeq platform (San Diego, CA, USA) at Beijing Novogene Bioinformation Technology Co., Ltd. (Beijing, China).

First-strand cDNA was synthesized from 1 µg above total RNA using the Evo M-MLV RT Kit with gDNA Clean for qPCR II (Accurate Biology, Changsha, China). Quantitative real-time PCR (qRT-PCR) was performed on a Bio-Rad CFX Connect Real-Time PCR System using Tsingke TSE501 ArtiCanATM SYBR qPCR Mix (Tsingke Biotechnology, Beijing, China). The *Actin* gene from *P. sojae* was used according to Li et al. [28], and the primers used in this experiment are listed in Table S1. Relative sporangium formation-related genes and ABC transporter genes transcript levels were calculated using the 2^−∆∆CT^ method [29]. Means and standard deviations were obtained from three biological replicates.

## 3. Results

### 3.1. Sequence and Expression Profile Analyses of Ps495620

**Figure 1:**
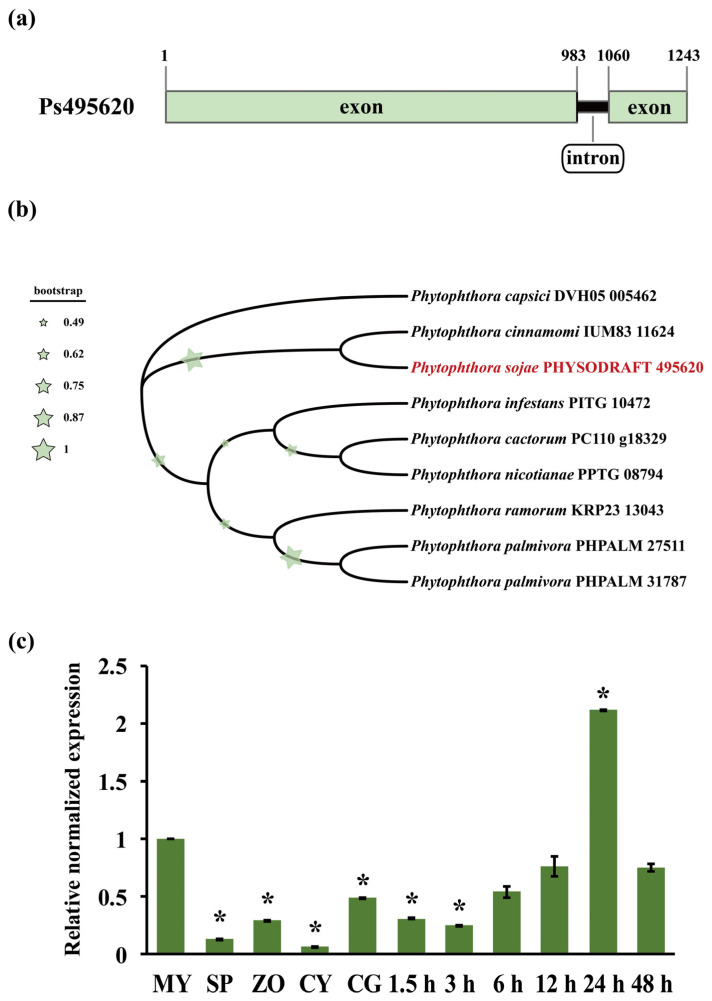
Sequence and expression profile analysis of Ps495620. (**a**) Full-length Ps495620 gene; (**b**) phylogenetic tree of Ps495620 homologous proteins from *P. capsici*, *P. infestans*, *P. ramorum*, *P. palmivora*, *P. cinnamomi*, *P. nicotianae*, and *P. cactorum*, based on Bayesian inference. The size of a star of bootstrap stands for confidence in the branching of phylogenetic trees; (**c**) expression profile of Ps495620 at various developmental and infection stages, measured by qRT-PCR. Samples include mycelia (MY), sporangium (SP), zoospore (ZO), cystospores (CY), cyst germination (CG), and different infection stages of strain P6497 (at 1.5 h, 3 h, 6 h, 12 h, 24 h, and 48 h). Statistical significance was determined using one-way ANOVA, with asterisks indicating significant differences (*p* < 0.01).

*Ps495620* has a total length of 1243 bp and one intron (b. 1a). Homologous proteins were identified using Ps495620 as a query against oomycetes. Eight homologous proteins were identified: PHPALM_31787 and PHPALM_27511 in *Phytophthora palmivora*, IUM83_11624 in *Phytophthora cinnamomi,* PPTG_08794 in *Phytophthora nicotianae*, PC110_g18329 in *Phytophthora cactorum*, DVH05_005462 in *Phytophthora capsici*, KRP23_13043 in *Phytophthora ramorum*, and PITG_10472 in *Phytophthora infestans*. Phylogenetic analysis showed that proteins homologous to Ps495620 are distributed across different clades, suggesting functional divergence (Figure 1b). *Ps495620* transcripts were found to be abundant in *P. sojae* at various developmental and infection stages. *Ps495620* transcripts were upregulated more than twofold during infection at 24 h compared to mycelia, indicating that *Ps495620* may play a key role in *P*. *sojae* infection. *Ps495620* expression decreased during the SP, ZO, CY, and CG stages (Figure 1c).

### 3.2. Ps495620 Plays a Critical Role in Sporangium Formation and Oospore Production

**Figure 2:**
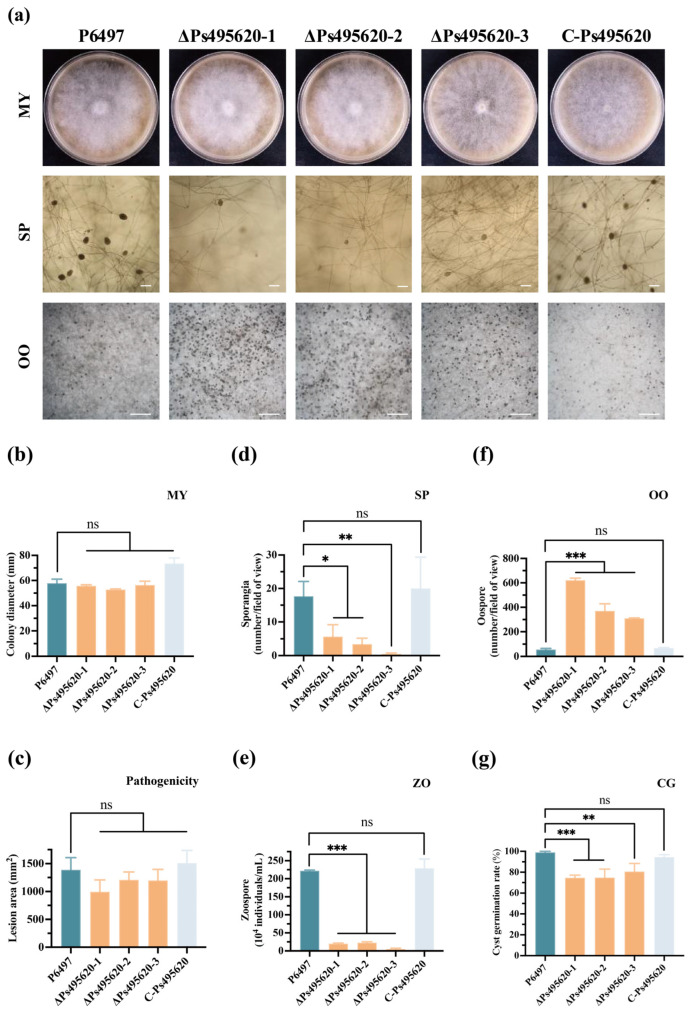
Phenotypic analysis of *Ps495620* knockout transformants. (**a**) Colony diameter (first row) on V8 medium, with microscopic visualization of sporangia (second row) and oospores (third row) of wild-type strain P6497 (WT), *Ps495620* knockout transformants (∆Ps495620-1, 2, 3) and complemented transformant (Ps495620-C); (**b**) colony diameter on V8 medium; (**c**) pathogenicity on soybean leaves; (**d**) sporangia number; (**e**) zoospore number; (**f**) oospore number; (**g**) cyst germination. Experiments were repeated three times. ns: not significant. *: At *p* < 0.05, significant difference. **: At *p* < 0.01, significant difference. ***: At *p* < 0.001, significant difference. The scale bar represents 50 µm.

In order to investigate the biological function of Ps495620, knockout transformants were generated using the CRISPR/Cas9-mediated gene editing system and validated via PCR, producing the lines ΔPs495620-1, ΔPs495620-2, and ΔPs495620-3 (Figure S2). The complemented transformant C-Ps495620 strain was obtained by overexpressing full-length *Ps495620* in ΔPs495620-1. Compared to the WT strain, *Ps495620* knockout mutants showed no significant differences in mycelial growth (Figure 2a,b) or pathogenicity (Figure 2c). However, the *Ps495620* knockout transformants exhibited significant reductions in sporangium and zoospore production (Figure 2a,d,e) and significantly increased oospore production (Figure 2a,f) compared to the WT strain. While deleting *Ps495620* did not affect mycelial growth, it reduced cyst germination by approximately 10% (Figure 2a,g). Phenotypic variation was restored in the C-Ps495620 strain. These results indicate that *Ps495620* is essential for both sexual and asexual reproduction in *P. sojae*.

### 3.3. Ps495620 Is Crucial for the Stress Response in P. sojae

**Figure 3:**
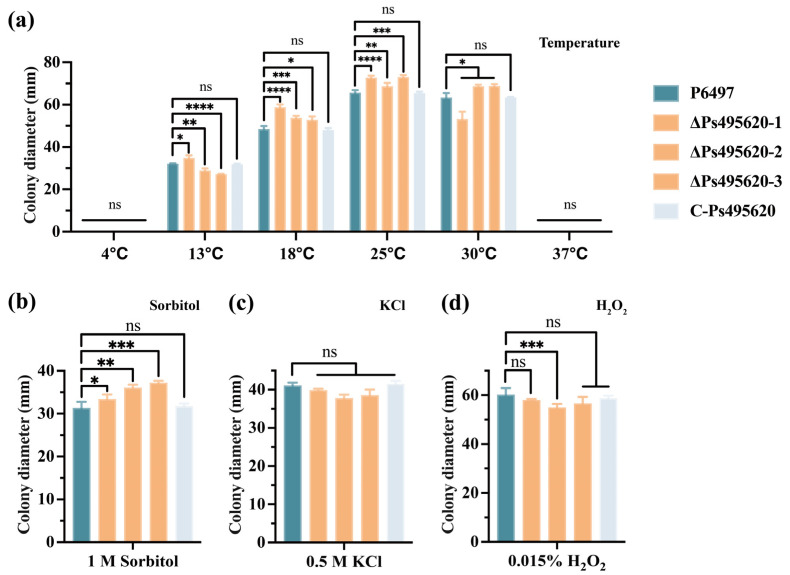
Phenotypic analysis of *Ps495620* knockout transformants under various stress conditions. (**a**) Mycelial growth under temperature stress; (**b**,**c**) mycelial growth under osmotic stress (sorbitol and KCl); (**d**) mycelial growth under oxygen stress. A one-way ANOVA was used for statistical analysis. *: At *p* < 0.05, significant difference. **: At *p* < 0.01, significant difference. ***: At *p* < 0.001, significant difference. ****: At *p* < 0.0001, significant difference. ns, not significant.

The growth of *Ps495620* knockout transformants was significantly different from the WT strain under various temperature conditions (Figure 3a). Similarly, under 1 M Sorbitol osmotic stress conditions, mycelial growth on Plich medium was significantly different between *Ps495620* knockout mutants, the WT strain, and the C-Ps495620 transformant (Figure 3b). However, under 0.5 M KCl osmotic stress conditions, ΔPs495620 did not differ significantly in mycelial growth on Plich medium between *Ps495620*-knockout mutants, WT, and C-Ps495620 transformant (Figure 3c). Additionally, exposure to 0.015% H_2_O_2_ did not result in significant differences in mycelial growth between the *Ps495620* knockout mutants and control strains (Figure 3d). These results suggest that *Ps495620* is required for responses to temperature and osmotic stress in *P. sojae*.

### 3.4. Transcriptome Analysis of Ps495620 Knockout Transformants at the Sporangia Stage

**Figure 4:**
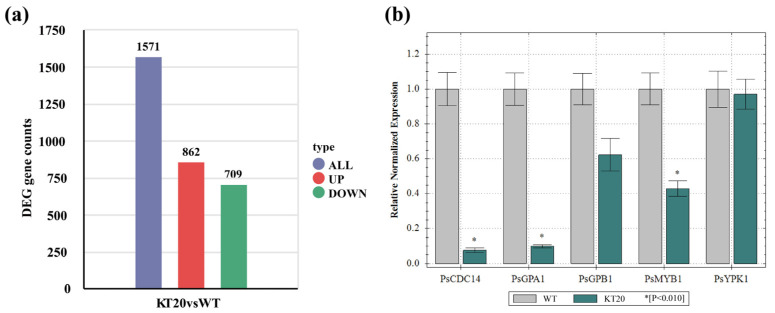
Transcriptomic analysis of *Ps495620* knockout transformants and P6497 (WT) in sporangia stage. (**a**) Volcanic diagrams displayed the differentially expressed genes (DEGs) among Ps495620 knockout transformants (KT20) and P6497 (WT) in sporangia stage; (**b**) differential gene expression analysis of *Ps495620* knockout transformants and P6497 (WT) in sporangia stage, measured by qRT-PCR. *: At *p* < 0.01, significant difference.

To investigate the regulatory role of Ps495620 in gene expression during the sporangia stage, a transcriptomic analysis of *Ps495620* knockout transformants (ΔPs495620-1, KT20) and the WT strain P6497 was performed. A comparison between ΔPs495620 and P6497 (WT) identified 1571 differentially expressed genes, with 862 upregulated and 709 downregulated (Figure 4a). Previous studies have associated the serine/threonine protein kinase *PsYPK1* (PHYSODRAFT_350990), the G protein α subunit *PsGPA1* (PHYSODRAFT_322123) [30], the G protein β subunit *PsGPB1* (PHYSODRAFT_301543) [12], the cell cycle regulator *PsCDC14* (PHYSODRAFT_515577) [31], and the MYB transcription factor *PsMYB1* (PHYSODRAFT_350123) [32] with sporangium formation in *P. sojae*. We performed qRT-PCR analysis to explore the expression of these genes in ΔPs495620 and P6497 (WT) at the sporangia stage. Among these genes, *PsCDC14*, *PsGPA1*, and *PsMYB1* were downregulated, while *PsGPB1* and *PsYPK1* showed no significant changes compared to the WT strain (Figure 4b). The expression profiles obtained by qRT-PCR were consistent with the RNA-seq results, confirming the reliability of the RNA-seq data.

**Figure 5:**
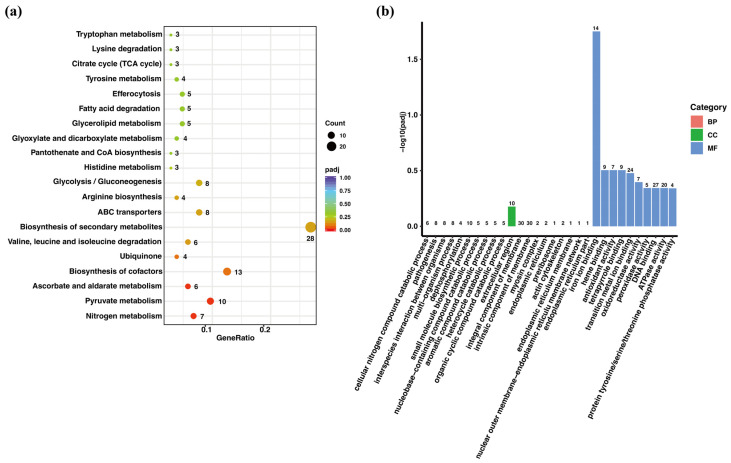
Differential gene expression enrichment analysis of *Ps495620* knockout transformant and P6497 (WT) in sporangia stage. (**a**) KEGG functional enrichment analysis; The size of the circle stand for the count of genes, and the change in the color of the circle stand for *p*-adjusted (padj); (**b**) GO functional enrichment analysis; red bars represent biological process (BP), green bars represent cellular component (CC), and blue bars represent Molecular Function (MF). The numbers on top of each bar indicate the number of genes involved in each process.

Kyoto Encyclopedia of Genes and Genomes (KEGG) pathway analysis revealed that the differentially expressed genes were involved in various metabolic processes, including nitrogen, pyruvate, ascorbate, and aldarate metabolism; the biosynthesis of cofactors, ubiquinone, and other terpenoid-quinones; valine, leucine, and isoleucine degradation; the biosynthesis of secondary metabolites; ATP-binding cassette (ABC) transporters; arginine biosynthesis; glycolysis/gluconeogenesis; and histidine metabolism. These findings suggest that Ps495620 plays a critical regulatory role in the metabolic processes of *P. sojae* (Figure 5a).

Gene ontology (GO) analysis of ΔPs495620-1 highlighted significant enrichment in the cellular component (CC) category, particularly in the extracellular region. Molecular function (MF) enrichment analysis showed predominant activities related to iron ion binding, heme binding, antioxidant activity, tetrapyrrole binding, transition metal ion binding, oxidoreductase activity (acting on paired donors with incorporation or reduction of molecular oxygen), peroxidase activity, DNA binding, ATPase activity, and protein tyrosine/serine/threonine phosphatase activity (Figure 5b). Notably, ABC transporters were enriched in both the KEGG and GO analyses. In the KEGG enrichment analysis, these ABC transporters were associated with ATPase activity, which was also highlighted in the GO enrichment analysis.

### 3.5. Differential Gene Expression Analysis of Ps495620 Knockout Transformant (KT20) and P6497 (WT) Strains at the Sporangia Stage

**Figure 6:**
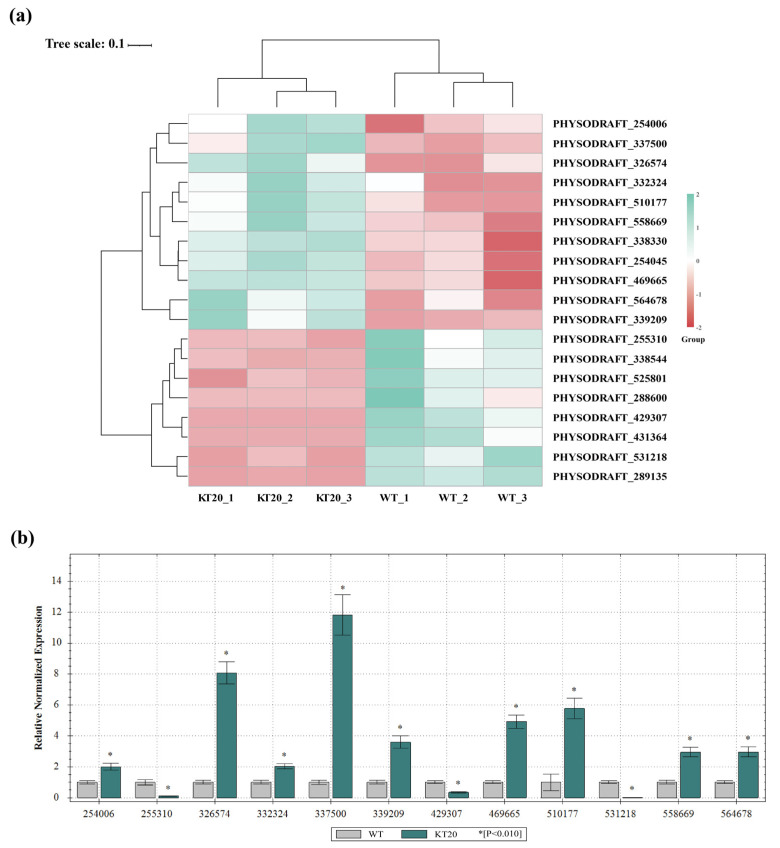
Differential ABC transporter genes expression analysis of *Ps495620* knockout transformants and P6497 (WT) in sporangia stage. (**a**) Clustering heat map of differentially expressed ABC transporter genes among *Ps495620* knockout transformants (KT20) and P6497 (WT) in KEGG and GO enrichment analysis. Three to four biological replicates at different time intervals were used for RNA-seq analysis. The color gradient represents the relative sequence abundance; numbers in the color key indicate log2 fold change (FC); (**b**) expression profile of ABC transporter proteins of *Ps495620* knockout transformants and P6497 (WT) in sporangia stage, measured by qRT-PCR. *: At *p* < 0.01, significant difference.

**Figure 7:**
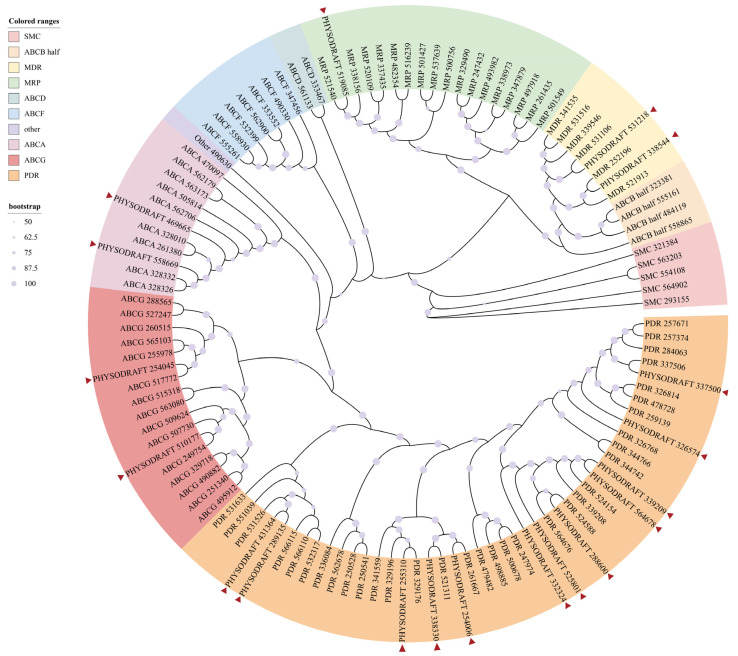
Phylogenetic tree of differential ABC transporter proteins expression analysis of *Ps495620* knockout transformants and P6497 (WT) in sporangia stage in *Phytophthora sojae*. The colored ranges stand for the kinds of ABC transporter proteins. The size of a circle of bootstrap stands for confidence in the branching of phylogenetic trees. The red triangles stand for ABC transporter proteins from RNA-seq of *Ps495620* knockout transformants and P6497 (WT) in sporangia stage.

ABC transporters are a large superfamily of proteins ubiquitously present in all living organisms. They are classified into seven subfamilies, ABCA to ABCG, based on gene and protein sequence homology, with each subfamily performing distinct functions, such as transporting lipids, drugs, peptides, and ions [33,34]. We identified 19 significantly differentially expressed ABC transporters between the KT20 and WT strains, with 11 upregulated and 8 genes downregulated. Heatmaps were generated based on the RNA-seq data to visualize the expression changes in sporangia-related genes (Figure 6a). In order to validate these transcriptomic findings, 12 ABC transporters with significant expression changes were selected for qRT-PCR validation. In the qRT-PCR analysis, nine were upregulated, and three were downregulated (Figure 6b), consistent with the RNA-seq data. Additionally, a phylogenetic tree was constructed for further analysis of the diversity of these ABC transporters. It revealed the presence of ABCA, ABCB (multidrug resistance [MDR]), ABCC (MDR-associated protein [MRP]), and ABCG (pleiotropic drug resistance [PDR]) members within the ABC transporter superfamily (Figure 7).

## 4. Discussion

We identified eight homologs of Ps495620, which showed over 60% sequence similarity, suggesting a conserved functional role in oomycetes. The expression profile of Ps495620 indicated that it may be associated with the pathogenicity of *P. sojae*. Interestingly, the ΔPs495620 knockout transformant showed no significant differences in pathogenicity. However, the observed reduction in sporangium and zoospore formation and increased oospore production suggests that Ps495620 is likely involved in regulating developmental processes related to both sexual and asexual reproduction in *P. sojae*. *Ps495620* was downregulated during the SP and ZO stages, contradicting the observed reduction in sporangium and zoospore formation in *Ps495620* knockout mutants. Multiple biological mechanisms might explain the discrepancy between the expression patterns of *Ps495620* and the observed phenotypes after its knockout. They include: (a) changes in gene expression do not always correspond to changes in protein activity since the latter can also be regulated by post-translational modifications (e.g., phosphorylation and methylation) [35]; (b) epigenetic modifications such as DNA methylation and histone modifications can regulate gene expression without altering mRNA levels [36]; (c) *Ps495620* may encode non-coding RNAs that play a role in gene regulatory networks, affecting the expression of other genes [37]; (d) Ps495620 may be involved in complex regulatory networks, including feedback inhibition or signaling pathway crosstalk, where knocking out *Ps495620* triggers secondary effects, leading to phenotypic changes that do not correlate with its expression patterns.

Transcriptomic data from ΔPs495620-1 suggest that Ps495620 plays a key regulatory role in metabolic processes, particularly nitrogen, pyruvate, and ascorbate metabolism. GO analysis shows that genes differentially expressed between the ΔPs495620-1 and WT strains are primarily involved in metabolism and biosynthesis, particularly of small molecules and organic acids, as well as enzyme catalytic and binding activities. The significant enrichment of genes related to small molecule metabolism highlights their role in metabolism, energy production, and signal transduction. Notably, ABC transporters were identified in both the KEGG and GO enrichment analyses. The transcriptomic data identified ABC transporters belonging to several subfamilies, including ABCA, ABCB (MDR), ABCC (MRP), and ABCG (PDR).

MDR members of the ABCB subfamily are involved in drug efflux, peptide transport, and bile acid secretion. For example, ABCB1 is located on the cell membrane and uses ATP hydrolysis to transport various compounds, including chemotherapeutic agents, out of cells, leading to MDR in tumor cells [38]. ABCB10 is found on the inner mitochondrial membrane and is involved in heme biosynthesis and erythropoiesis. *ABCB10* is highly expressed in early erythroid cells and may protect mitochondria from oxidative damage. In plants, the MDR transporters are localized on the plasma membrane and function as efflux transporters: MDR transporters in *Coptis japonica* facilitate the active uptake of berberine by xylem vessels [39], and At2g36910 and At3g28360 regulate auxin transport in *Arabidopsis* [40].

The MRP members of the ABCC subfamily are involved in transporting organic anions, drug metabolites, and ions. For example, At2g34660 regulates anthocyanins in *Arabidopsis* [41], while ABCC8 (SUR1) and ABCC9 (SUR2) regulate K^+^ channels in vertebrates [42].

The ABCA subfamily primarily participates in the transmembrane transport of lipids, including cholesterol, phospholipids, and other lipid molecules, affecting the composition and function of cell membranes. ABCA1 transfers cholesterol and phospholipids to apolipoprotein A1 (ApoA1), promoting the formation of high-density lipoprotein (HDL), which is involved in reverse cholesterol transport [43]. ABCA3 is expressed in pulmonary type II alveolar cells and is involved in phospholipid transport for pulmonary surfactant, maintaining normal lung function [44].

The PDR members in the ABCG subfamily are involved in transporting cholesterol, bile acids, steroid hormones, drugs, and xenobiotics. ABCG5 and ABCG8 form a heterodimer, limiting the absorption of plant sterols in the intestine and promoting the secretion of cholesterol from the liver and intestine into bile and the intestinal lumen [45]. ABCG1 promotes the transport of cholesterol and phospholipids from cells to HDL, involved in cholesterol efflux [46].

In our transcriptomic analysis, the upregulation of ABCA, ABCG, and MRP members suggests that the lack of Ps495620 affects the transmembrane transport of lipids and ions. Conversely, MDR members were downregulated, affecting peptide transport, while PDR members were both upregulated and downregulated, indicating dysregulated steroid transport.

We measured the expression of selected genes in the *Ps495620* knockout transformants and the P6497 (WT) strain at the sporangia stage using qRT-PCR. Among them, *PsCDC14*, *PsGPA1*, and *PsMYB1* were downregulated, while *PsGPB1* and *PsYPK1* showed no significant changes compared to the WT strain. These results suggest that Ps495620 regulates sporangium production independent of the PsGPB1 and PsYPK1 pathways, possibly affecting the expression of *PsCDC14* and *PsMYB1* through upstream regulatory pathways. The lack of Ps495620 resulted in the downregulation of *PsGPA1*, impairing its signaling response.

## 5. Conclusions

In our study, oospore production was significantly increased in the *Ps495620* knockout transformants compared to the WT strain, and we observed significant differences in the expression of 19 ABC transporters. Since the oospore is composed of lipids, proteins, and β-glucans [4], Ps495620 likely influenced the transport of these components, potentially explaining increased oospore production. In conclusion, our study identified Ps495620 as a key regulator of reproductive and metabolic processes in *P. sojae*. Future studies should focus on elucidating the molecular mechanisms through which Ps495620 regulates these pathways, using protein interaction and structural analyses to deepen our understanding of its role in the metabolic networks of *P. sojae*. Based on its unique sequence characteristics, no highly similar homologous proteins have been identified in non-oomycete organisms. Given its critical role in the formation of sporangia in *Phytophthora sojae*, this protein exhibits potential as a molecular target for novel fungicides. Future studies could focus on elucidating the structure of Ps495620 and employing computer-assisted drug design to develop innovative oomycete inhibitors.

## Data Availability

The original contributions presented in the study are included in the article/Supplementary Materials, further inquiries can be directed to the corresponding authors.

## Supplementary Materials

The following supporting information can be downloaded at: https://www.mdpi.com/article/10.3390/jof11010012/s1, Figure S1: Bioinformatic analysis of Ps495620; Figure S2: CRISPR/Cas9-mediated Ps495620 gene knockout; Table S1: Primer used in this study.
