# Supplementary material for: A Protein with Unknown Function, Ps495620, Is Critical for the Sporulation and Oospore Production of Phytophthora sojae
Source: J Fungi (Basel). 2024 Dec 27;11(1):12. doi: 10.3390/jof11010012 (PMC11766772; doi:10.3390/jof11010012)
Supplement: Supplementary file 1 [file jof-11-00012-s001.zip › jof-3363404-supplementary.pdf]

(a)

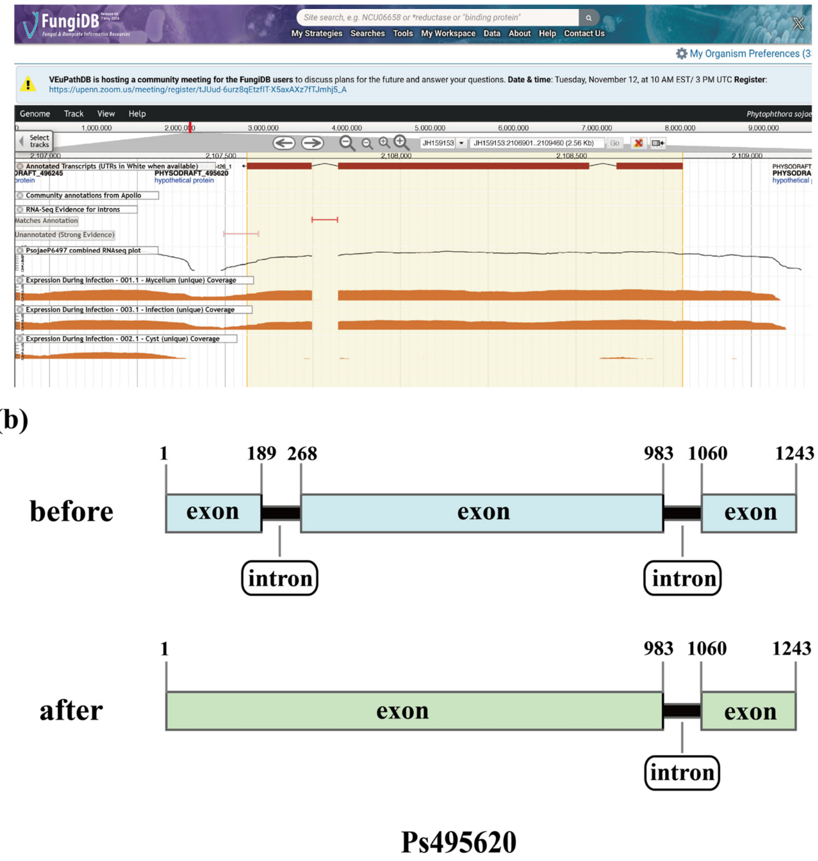

Figure S1. Bioinformatic analysis of Ps495620. (**a**) RNA-seq data from the FungiDB database (https://fungidb.org/fungidb/app). (**b**) Schematic plot of prediction and verified gene sequence for Ps495620.

(a)

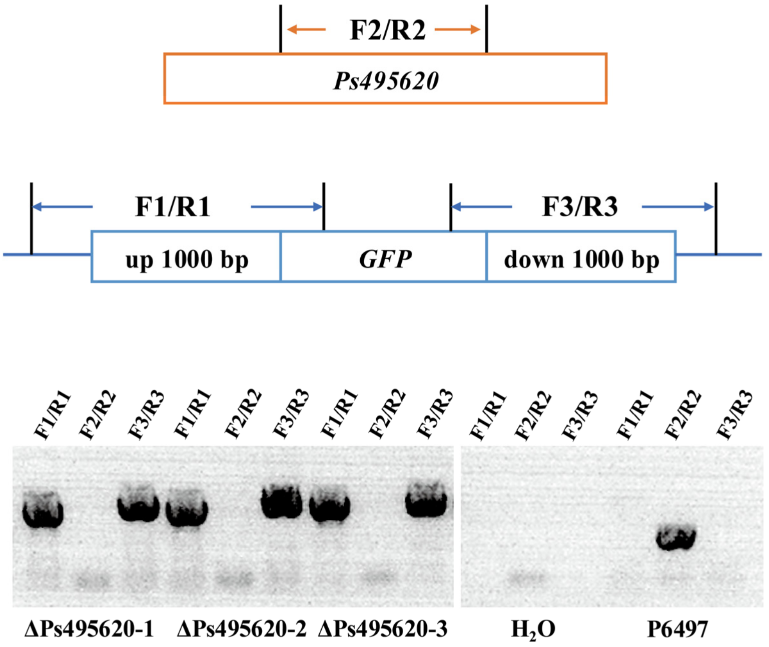

Figure S2. CRISPR/Cas9-mediated Ps495620 gene knockout. (**a**) Primer locations for transformant screening are indicated by the arrows. (**b**) PCR results demonstrated that Ps495620 was completely replaced by the *GFP* gene P6497, wild-type; ΔPs495620-1, ΔPs495620-2, and ΔPs495620-3, Ps495620 knockout mutants.

Table S1. Primer used in this study.

Primer name	Primer sequence (5'-3')	Application
PsActin-F	ACTGCACCTTCCAGACCATC	Reference gene primer
PsActin-R	CCACCACCTTGATCTTCATG	
Ps495620-qPCR-F	AGTCCAGTGGCGTGGGTG	Real-time quantitative PCR analyze
Ps495620-qPCR-R	TGCCGTCTCTGTCTCGAC	
Ps495620-mCherry-F	TATCGATAGGCCTCCGCGGATGACGGCGGAGTGGCAG	Construction of *Pc495620* overexpression vector
Ps495620-mCherry-R	CTGCCTCCTCCACCGGGCCCAATGCTCTTGAGGAAGAGCGGAG	
Ps495620-sgRNA1-F	CTAGCTTGTCCCTGATGAGTCCGTGAGGACGAAACGAGTAAGCTCGTCGGACAACCGCAGACTCGACG	Construction of *Pc495620* knockout vector
Ps495620-sgRNA1-R	AAACCGTCGAGTCTGCGGTTGTCCGACGAGCTTACTCGTTTCGTCCTCACGGACTCATCAGGGACAAG	
Ps495620-sgRNA2-F	CTAGCCAACCTCTGATGAGTCCGTGAGGACGAAACGAGTAAGCTCGTCAGGTTGCAGGGATAGATAGC	
Ps495620-sgRNA2-R	AAACGCTATCTATCCCTGCAACCTGACGAGCTTACTCGTTTCGTCCTCACGGACTCATCA GAGGTTGG	
Ps495620-Donor-F1	ATCGATAAGCTTGATATCGAATTCGTCCTTCGACGAGTCCAT	
Ps495620-Donor-R1	TTCCTCGCCCTTGCCCATGGCCTCGAGAGTCGGGGA	
Ps495620-Donor-F2	TCCCCGACTCTCGAGGCCATGGGCAAGGGCGAGGAA	
Ps495620-Donor-R2	GTTAGCCCAGTCAACGCGTTCCGGAGTT	
Ps495620-Donor-F3	AACGCGTTGACTGGGCTAACTGTAAGCTCATGATG	
Ps495620-Donor-R3	CGCTCTAGAACTAGTGGATCCGAGCTTGCGGAAGCAGAAGATC	
Ps495620-QCYZ-F1	CGCAGCCTGTCGGACGAG	Transformant screening
Ps495620-QCYZ-R1	GTGAAAGTGGTGACGAGGGTTG	
Ps495620-QCYZ-F2	GCCACTCCCGCCTCTGTC	
Ps495620-QCYZ-R2	AATACGACCAGCACCGATAGTTC	
Ps495620-QCYZ-F3	CGGACCACTACCAGCAGAACAC	
Ps495620-QCYZ-R3	CGCACGCTTTACTTGATGAAGAGG	
Ps564678-qPCR-F	GTACTCGCCGAAACAC	Real-time quantitative PCR analyze
Ps564678-qPCR-R	GAACGGGAAGATCAACG	
Ps558669-qPCR-F	ATTGCCTCTGACGACTC	
Ps558669-qPCR-R	CATTGCTACGCTGTATCC	
Ps531218-qPCR-F	ATCAGCGAGCACTTGGAC	
Ps531218-qPCR-R	GATGAGGACAGCGGAACC	
Ps510177-qPCR-F	ACTCGCTGCTCGGGTC	
Ps510177-qPCR-R	GATGAGGATGCTCGGGTTC	
Ps469665-qPCR-F	ACTCGTCGTCACCTTAC	
Ps469665-qPCR-R	GGCATCCTCCGTATCG	

Ps429307-qPCR-F	AGCGCTTCTACAAGAAGT
Ps429307-qPCR-R	ACGGCGTGAAGATGGAGG
Ps339209-qPCR-F	ACCGGCATCCTCTTCA
Ps339209-qPCR-R	AGCTCGTCGCAGTTGGC
Ps337500-qPCR-F	GCGTCCTCCCTTTCAC
Ps337500-qPCR-R	AGAGATGTAGTAGCACCAC
Ps332324-qPCR-F	CTGACGATGAAGGCGACTACC
Ps332324-qPCR-R	GGCTTAGGCACCGAGTAACG
Ps326574-qPCR-F	CCCACCAATGTCCAAG
Ps326574-qPCR-R	ATGTAGCAGATGAAGGC
Ps255310-qPCR-F	CGTGGCGTTCAATGGTTC
Ps255310-qPCR-R	GGCGGCTTGGTTCTCC
Ps254006-qPCR-F	ACGGCCAATCAGCACAC
Ps254006-qPCR-R	GACCTCGCCCTCGATCGA
PsYPK1-qPCR-F1	TTCCTCGTCCGCAAGAAG
PsYPK1-qPCR-R1	TCCGCCAGAGCAATAGTC
PsCDC14-qPCR-F	CTCGGCGATGGCAAACGG
PsCDC14-qPCR-R	CGGAGAAGACGAATGTGTGG
PsMYB1-qPCR-F	TGAACCAGAGCAACCAGAATG
PsMYB1-qPCR-R	ATGACAACCGACCCAGGAC
PsGPA1-qPCR-F	CGTCCCAGATGAGCGATG
PsGPA1-qPCR-R	CGTTGTTATAGATAATTGGTGTGC
PsGPB1-qPCR-F	GCACCACGGGCTCACAC
PsGPB1-qPCR-R	CGAGGCAAGATACACGGTTC

.
